# Nanowires for 2D material-based photonic and optoelectronic devices

**DOI:** 10.1515/nanoph-2021-0800

**Published:** 2022-02-08

**Authors:** Ha Young Lee, Sejeong Kim

**Affiliations:** Department of Electrical and Electronic Engineering, Faculty of Engineering and Information Technology, University of Melbourne, Victoria 3010, Australia

**Keywords:** 2D materials, integrated photonic circuits, nanophotonics, nanowires, optoelectronics

## Abstract

Nanowires have garnered considerable attention in photonics and optoelectronics due to their unique features. Owing to the large surface area and significant potential of usage as a resonator and waveguide in photonic integrated circuits (PICs), nanowires have been applied in many research areas in nanophotonics. To enhance the properties of light emitting materials, the hybrid of nanowires and 2D materials has been deployed in many papers. This paper summarises recent studies on the application of various types of nanowires in photonics and optoelectronics, as well as the combination of nanowires and 2D materials. This review article introduces nanowires that act as resonators or/and waveguides to increase the performance of 2D materials used in PICs for light enhancement and guiding. Moreover, the review lays out the hybrid of nanowires and 2D materials that have been studied in the field of optoelectronics. The hybridization of nanowires and 2D materials for photonics and optoelectronics is discussed in this review for the outlook of future studies.

## 1 Introduction

The study of the interaction of light and matter has gained popularity for several decades. Recent emphasis has been focused on improving the strength of light–matter interaction in order to realise compact integrated photonic circuits [1], [2], [3], efficient photonic devices [4, 5], and multi-functioning optoelectronic systems [6, 7]. 2D materials are one of the most vigorously investigated materials in modern science. There are various advantages of using 2D materials for research. For example, 2D materials provide favorable mechanical properties such as being highly bendable and stretchable without causing damage [8]. Additionally, one can easily create atomically smooth, mono- or few layered samples by simply exfoliating 2D materials from the bulk crystal using a sticky tape, which has increased the use of 2D materials for laboratory study. Through the exfoliation method, 2D materials can be transferred or stacked onto any materials without regard for lattice-mismatch concern. So far, researchers have identified a library of 2D materials with characteristics ranging from metals to insulators, and these materials sometimes exhibit unique properties such as high electrical conductivity [9], high nonlinearity [10, 11] or valley-dependent electrical/optical response [12].

**Figure 1:**
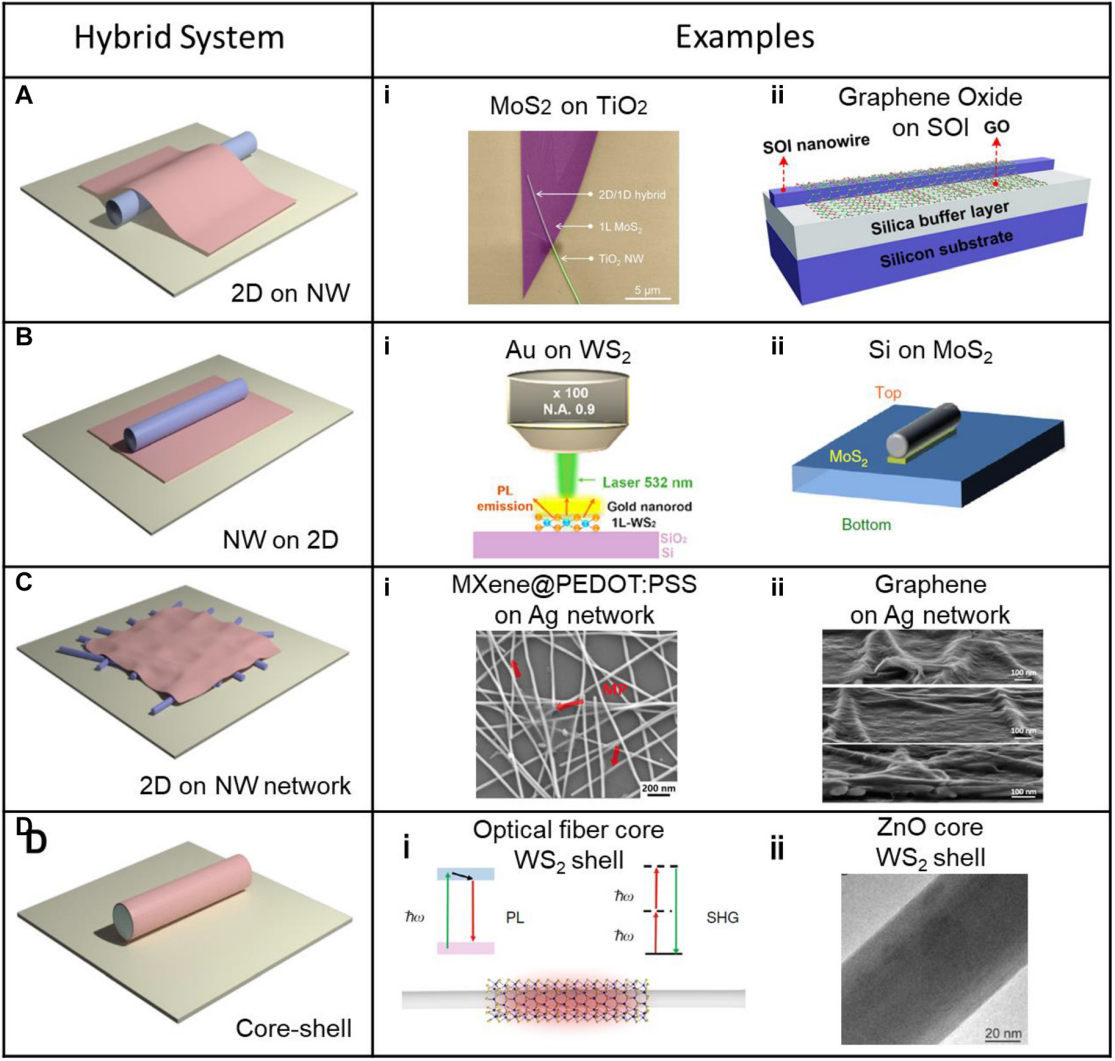
Schematic of the hybridization of nanowires and 2D materials. (A) 3D structure diagram of a 2D on a nanowire. (A-i) False-color SEM image of a monolayer MoS_2_/NW hybrid structure. Reproduced/adapted with permission from Li et al. [21]. Copyright 2019 American Chemical Society. (A-ii) Schematic of a GO-coated on an SOI nanowire waveguide. Reproduced/adapted with permission from Zhang et al. [22]. Copyright 2020 American Chemical Society. (B) 3D structure diagram of a nanowire on a 2D material. (B-i) Schematic showing photoluminescence measurement of a gold nanorod on a monolayer WS_2_ heterostructure. Reproduced/adapted with permission from Jiang et al. [23]. Copyright 2020 American Chemical Society. (B-ii) Schematic of a silicon nanowire on a MoS_2_ layer. Reproduced/adapted with permission from Cihan et al. [19]. Copyright 2018 Macmillan Publishers Limited, part of Springer Nature. (C) 3D structure diagram of a 2D material covering a nanowire network. (C-i) SEM image of AgNW-MXene@PEDOT:PSS hybrid. Reproduced/adapted with permission from Bai et al. [24]. Copyright 2020 Elsevier Ltd. (C-ii) SEM images of EG thin layer on AgNWs network. Reproduced/adapted with permission from Ricciardulli et al. [25]. Copyright 2018 Wiley-VCH Verlag GmbH & Co. KGaA, Weinheim. (D) 3D structure diagram of a nanowire-core/2D material-shell. (D-i) Schematic of a hybrid WS_2_-optical-fiber-nanowire. Reproduced/adapted with permission from Chen et al. [26]. Copyright 2019 by the original authors (CCBY). (D-ii) TEM image of ZnO/WS_2_ nanowire annealed in sulfur atmosphere and additionally annealed in an inert atmosphere. Reproduced/adapted with permission from Butanovs et al. [27]. Copyright 2018 American Chemical Society.

The hybridization of nanowires with 2D materials enables 2D materials to function better as a photonic and electrical device. Nanowires can be made of metals, semiconductors or insulators. Metal nanowires are versatile since they can be used as both electrodes and photonic components. Silver is frequently adopted as an electrode material due to its high transmittance, low sheet resistance and high flexibility. By incorporating 2D materials such as MXene, graphene, or graphene oxide [13], one can address some of the bottlenecks that impeding their practical usage. For example, 2D conducting layers connects nanowires and smooth out the surface, resulting in reduced resistance. In addition, 2D insulating materials protect metal nanowires from oxidation. These heterostructures can be in various configurations as described in Figure 1. Besides electrodes, metal nanowires act as waveguides [14], open nanocavities [15], and control light emission properties [16]. As semiconductor manufacturing technology progresses [17], semiconductor nanowires are widespread and play an important role as a platform for integrated photonic circuits [18]. One significant advantage of semiconductor nanowires is their compatibility with complementary metal–oxide–semiconductor (CMOS) technology while yet providing advanced electrical and optical functionality [19, 20]. When these nanowires are combined with 2D materials in the form of core–shell or nanowire-on-monolayer structures, a synergetic effect is anticipated.

2D materials are also being investigated in quantum photonics as a promising quantum light source. For example, 2D semiconducting material such as WS_2_ and MoS_2_ monolayers and 2D insulating material such as hexagonal boron nitride (hBN) have single photon emitters. By incorporating nanowires, one can control the single photon emission and route it through the nanowires. 2D materials are also used to study second-harmonic generation (SHG) [21], and reduction of undesired emission is reported [19] by implementing semiconductor nanowires. Additionally, WS_2_ monolayers are widely used on photodetectors. The hybrid of the WS_2_ layer with ZnO [27] or CdS [28] nanowire was proven to have higher responsivity, faster operation, and higher absorption of light through band alignment induced charge carrier transfer. Also, perovskite nanowires including CsPbBr_3_ have high potentials for application in piezotronic [29] and piezo-phototronic devices [30, 31]. Moreover, their optical properties could be enhanced when incorporated with transition metal dichalcogenides (TMDs) materials. This is by enabling effective charge carrier transfer through the WS_2_ conducting channel while also suppressing dark current. WS_2_ monolayer is also integrated with optical fiber for enhancement of SHG by evanescent field coupling [26].

This article summarises the role of micro/nanowires in photonics and electronics. This article first discusses the use of nanowires as electrodes. Nanowires are advantageous as electrodes due to their high conductivity and flexibility. Then we expand the discussion to include their applications in photonics and electronics. Nanowires can operate as a photonic resonator and a waveguide, which aid in understanding how nanowires can enhance the functionality of photonics and electronics devices.

## 2 Conventional applications: nanowires as electrodes

Flexible and transparent electrodes are desirable for a variety of applications and are expected to find a wide range of usage in optoelectronics [32, 33]. Such electrodes have been used in flexible organic light-emitting diode (FOLEDs) [34, 35], solar cells [36], and many other optoelectronic applications [37, 38]. Metal nanowires are particularly attractive for the development of flexible transparent electrodes (FTE) due to their high transmittance and low sheet resistance [39, 40]. Traditionally, indium tin oxide (ITO) has been a widely adopted material for flexible and transparent electrodes. ITO has a high conductivity while being transparent at visible wavelengths. However, there are several drawbacks to employing ITO, including poor mechanical stability, which results in increased resistance due to cracks when bending the substrate [41]. Additionally, indium is a scarce raw material in the Earth’s crust, necessitating the use of substitute materials. Metal nanowires are promising candidates due to their exceptional optical and electrical properties [42, 43]. They demonstrated attractive features and are expected to replace ITO in commercial applications [44, 45]. This is because they are resilient to bending and folding cracks due to the increased elasticity provided by nanostructures while maintaining good electrical conductivity and optical transparency [46, 47].

However, metal nanowires retain certain inherent weaknesses, including high degree of surface roughness [48], low adhesion to the substrate, discontinuous structure between interface of nanowires [24] and fast degradation. These issues can be overcome by adding additional materials, i.e., creating a hybrid system. These hybrid systems consist of 2D materials with properties that are applicable in overcoming the problems. For example, MXene, a 2D material that consists of transition metal carbide, nitride, and carbonitride [49, 50], is frequently used to mitigate the problems. MXene is widely explored 2D material in the fields of sensors [51] and transparent electrodes [52] due to its features such as high electrical conductivity and large surface area. Graphene is also one of the promising 2D materials in this regard due to its unique electrical and optical properties [53].

**Figure 2:**
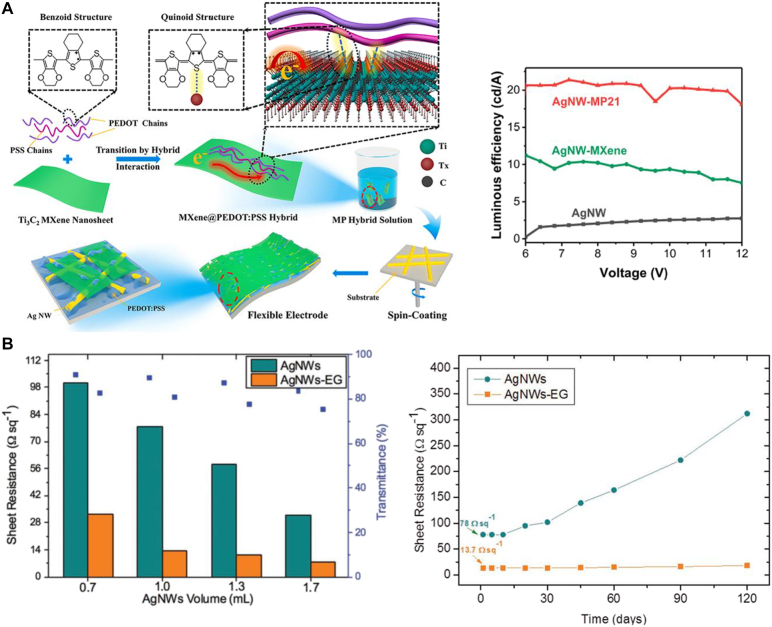
Integration of metallic NW and 2D materials for the flexible transparent electrodes. (A) Left, schematic illustration of the fabrication process of AgNW-MXene@PEDOT:PSS hybrid FTEs. Right, luminous efficiency of AgNW, AgNW-MXene hybrid, and AgNW-MP21 hybrid FTEs on silicon. Reproduced/adapted with permission from Bai et al. [24]. Copyright 2020 Elsevier Ltd. (B) Left, sheet resistance and transmittance of AgNWs and AgNWs-EG films at different AgNWs volumes. Right, long term sheet resistance of AgNWs and AgNWs-EG with air exposure. Reproduced/adapted with permission from Ricciardulli et al. [25]. Copyright 2018 Wiley-VCH Verlag GmbH & Co. KGaA, Weinheim.

Bai et al. [24] addressed issues relating to nanowire FTE by covering them with MXene, which effectively reduces the surface roughness of the nanowires. Using this approach, they achieved a low sheet resistance of 17 Ω/sq and high transmittance of 97.6% at *λ* = 550 nm. In detail, 2:1 mass ratio of Ti_3_C_2_T_*x*_ MXene and PEDOT:PSS hybrid solution (denoted as MP21) were diluted with DI water and stirred at room temperature for 2 h and it was spin-coated onto Ag nanowire FTE as shown in Figure 2A. As a result, MXene nanosheets covering Ag nanowire networks reduced surface roughness of Ag NW, and PEDOT:PSS polymer filling the hollow regions of Ag NW acted as a nanowelding process, reducing junction resistance. To investigate the light emitting performance and current efficiency of the sample, red FOLEDs were fabricated using the synthesized material. Compared to bare NW and NW-MXene, the Ag nanowire with MXene and PEDOT:PSS hybrid has improved on the aspect of luminance and current efficiency. The luminescence efficiency-voltage characteristics of Ag NW, Ag NW-MXene, and Ag NW-MP21 FTE based red FOLED were tested (Figure 2A). AgNW-MP21 based FOLEDs achieved the highest luminance efficiency of over 21 cd/A and a maximum external quantum efficiency (EQE) of 25.9%.

Similarly, graphene has been adopted to improve the conductivity of electrodes in hybrid systems. Ricciardulli et al. [25] demonstrated transparent electrodes composed of silver nanowires and electrochemically exfoliated graphene (EG). In detail, the solution containing silver nanowires is first sprayed onto flexible substrate, polyethylene naphthalate (PEN), followed by EG dispersion. In Figure 2B, sheet resistance and transmittance of various volume of silver nanowires with the EG layer are compared with the pristine Ag nanowires. Moreover, the samples were exposed in the air for 120 days for long-term stability (Figure 2B). During those times, the sheet resistance of the hybrid material remained constant while that of the pristine sample increased after 10 days of exposure. They reported that by deploying the EG layer, they were able to lower the sheet resistance (*R*_s_) without noticeable decrease of transmittance and simultaneously decreased the roughness from 78 Ω/sq to 13.7 Ω/sq and from 16.4 to 4.6 nm, respectively. Lowered sheet resistance and roughness was due to the EG coating as the dispersed layer flattened the surface of the Ag NW junctions and holes. This paper further demonstrated the implementation of the electrode as an anode in an organic solar cells (OSC) and polymer LEDs (PLEDs).

2D materials not only decrease the surface roughness, but also play an important role as a protective layer that prevents metal nanowires from oxidization. Silver nanowire, which is one of the most frequently used metal wires as a bottom electrode of perovskite solar cells (PVSC) has corrosion issues caused by the release of halide species from the perovskite layer [54]. Chen et al. [13] proposed the implementation of large-size graphene oxide (LGO) sheets as a protective layer for silver nanowire-based transparent electrodes. LGO sheets acting as protective layers are crucial to reduce the overall boundary area, as boundaries between the sheets lets in halide species. In this work, GO sheets with different sizes are separated by centrifugal method. The reduced LGO dispersion was dropped on the Ag NW electrode and dried using steady hot air flow. The electrode maintained its initial resistance for more than 45 h while the pristine sample exhibited exponential increase of resistance after 10 h at 0.8 V bias. This study demonstrates the possibility of constructing PVSC with high stability.

## 3 Nanowire as a versatile platform for nanophotonics

Nanowires offer a wide range of applications in photonics and electronics as they operate as a resonator and a waveguide. Mechanisms for nanowire working as a cavity and a waveguide are summarized in recent review paper by Z. Gu et al. [55]. Nanowires are Fabry–Perot (FP) type resonators that are composed of two mirrors that confine standing waves. In bulk optics, optical resonators are typically constructed using two mirrors with near-unity reflectivity (*R*); however, nanowires built of semiconductors or dielectric materials take use of the reflection induced by a change in the refractive index (*n*). The reflectivity at the interface of two materials having refractive index of *n*_1_ and *n*_2_ is determined by the Fresnel’s equation:

$$R={\left(\frac{n_{2}-n_{1}}{n_{2}+n_{1}}\right)}^{2}$$

The *Q*-factor (*Q*) of the FP type cavity is as follows:

$$Q=\frac{2\pi nL}{\lambda \left(1-R\right)}$$

Therefore, it is preferable to have a longer wire length (*L*) and a high refractive index at the incident wavelength (*λ*) to maximize light confinement. Numerous studies have been published in which nanowires have been used as the FP cavity. What’s noteworthy is that semiconductor nanowires are a complete nanolasers consisting of an optical resonator and a gain medium. Due to the ease of fabrication complexity in comparison to other type of nanolasers that requires photo or electron-beam lithography, semiconductor nanowires have been attracting considerable attention. Some of the recent lasing work includes lasing in cesium lead halide perovskite nanowires [56] and single mode lasing from CdS nanowires [57]. Apart from FP mode, microwires with a diameter in the micrometer range can allow whispering gallery mode (WGM) [58]. Therefore, an appropriate radius should be selected based on the optical modes to be used.

Another frequently adopted geometry for nanowire devices are nanowire-gap-substrate structure, which is used to facilitate gap mode. To achieve a gap mode, the gap layer is made of a thin dielectric material while nanowire or substrate, or both, are made of metal. This gap mode enables tight light focusing, which increases the Purcell factor, which is defined as the *Q*-factor divided by the mode volume. Therefore, nanowire-gap-substrate geometry is often used for nanowire lasing experiments. As an example, Xiang Zhang’s group [59] experimentally demonstrated a nanoscale plasmonic laser with optical mode smaller than the diffraction limit using CdS semiconductor nanowire, separated from a silver surface by an insulating gap.

Apart from the cavity effect, both dielectric and metallic nanowires have the potential to act as waveguides. Dielectric waveguides have a lower loss than metallic waveguides. Meanwhile, metallic nanowires on dielectric material or dielectric nanowire on metal substrate can be relatively lossy because they convey the light signal using surface plasmon polariton. The following section will cover nanowires for 2D materials. Nanowires can be used to increase the PL intensity of 2D TMDs via the cavity effect and to guide the emission from 2D materials via the waveguide effect. Additionally, the paper will outline a hybrid system composed of nanowires and two-dimensional materials for use as a quantum light source and photon detector.

## 4 Nanowires for 2D materials

### 4.1 Light enhancement

By increasing the spontaneous emission (SE) rate of radiative emission of light emitting 2D materials, a brighter light source can be created. The ratio of SE rate with and without a cavity is called Purcell factor, and it is proportional to *Q*-factor and inversely proportional to the optical mode volume. Many approaches have been made to achieve high PL intensity and this can be realized by using hybridization of nanowires with TMDs. Utilizing nanowires is also a popular approach for optical anisotropy [60, 61]. By tuning the morphology of the nanostructure, resonance frequency and quality factors could be controlled. With the incorporation of 2D TMDs with plasmonic or optical nanowires, effective control and enhancement of light could be made into practical use.

**Figure 3:**
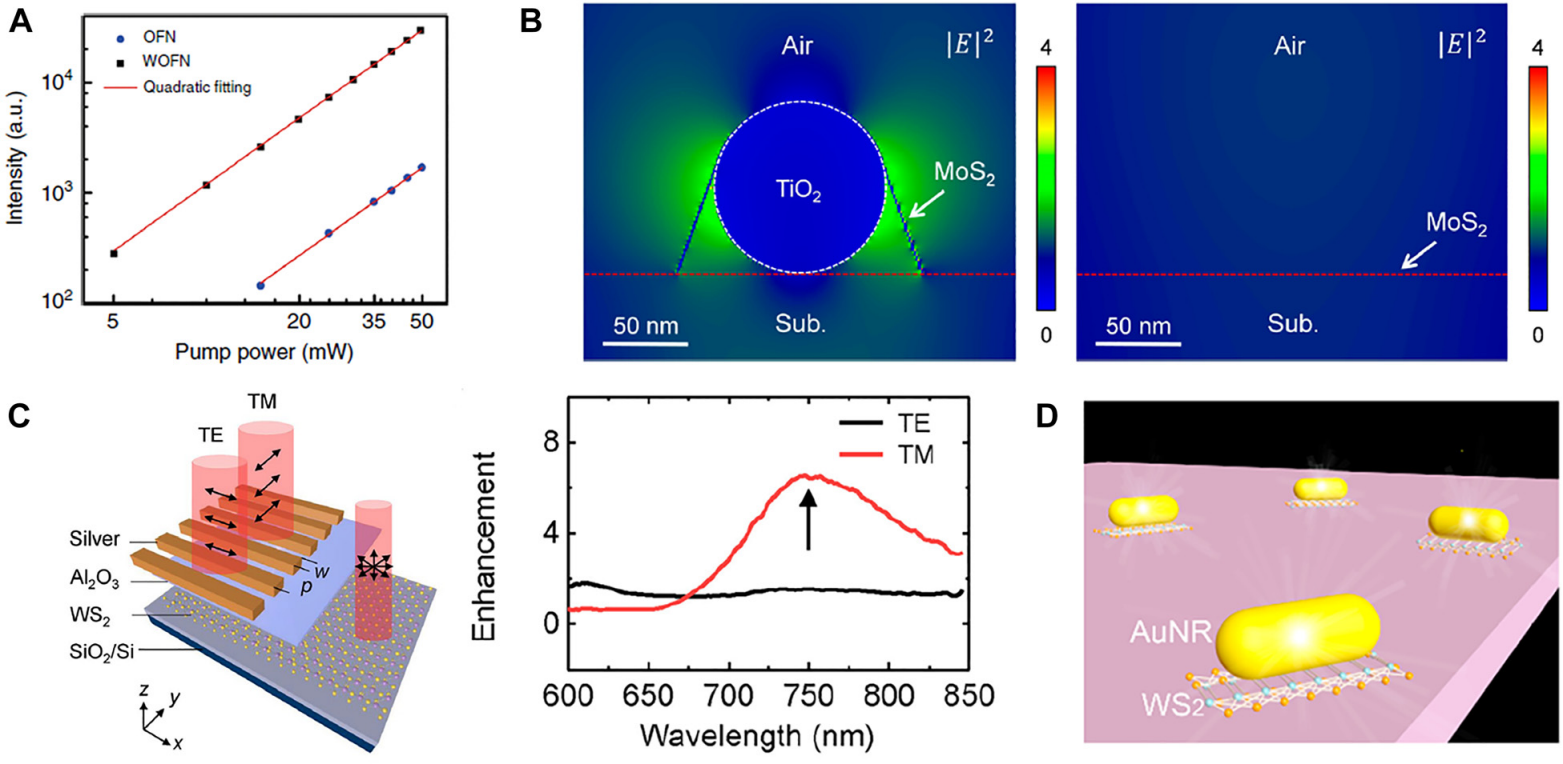
Hybrids of NW and 2D TMDs for the enhancement of light–matter interactions. (A) Second harmonic generation intensity as a function of the pump power for the OFN and WOFN. Reproduced/adapted with permission from Chen et al. [26]. Copyright 2019 by the original authors (CC BY). (B) Calculated electric field distribution (|E|^2^) excited by the fundamental wave (*λ* = 800 nm) in: MoS_2_/NW hybrid (left) and pristine MoS_2_ film (right). Reproduced/adapted with permission from Li et al. [21]. Copyright 2019 American Chemical Society. (C) Left, schematic of the hybrid device. Right, PL enhancements of the hybrid material relative to the pristine WS_2_ for TE and TM polarizations. Reproduced/adapted with permission from Han et al. [62]. Copyright 2021 by the original authors (CC BY). (D) Schematic of the heterostructure composed of a gold nanorod integrated on top of a WS_2_ monolayer. Reproduced/adapted with permission from Jiang et al. [23]. Copyright 2020 American Chemical Society.

The hybrids of nanowires and 2D TMDs are applied in various applications for the enhancement of light–matter interactions. Chen et al. [26] used a hybrid of optical fiber nanowire and WS_2_ for efficient second harmonic generation (SHG). Instead of the free-space coupling technique for SHG, the study employed an optical waveguide technique to enhance the conversion efficiency. The WS_2_-optical-fiber-nanowire (WOFN) was fabricated by laminating a WS_2_ monolayer on an OFN using a modified microtransfer technique. On Figure 3A, the SHG intensity as a function of pump power was measured. The WOFN showed a 20 times higher amount of the SHG intensity compared to that of the OFN. By the evanescent field coupling effect in the OFN, the enhancement of light–matter interaction can be expected.

Moreover, Li et al. [21] showed anisotropic enhancement of SHG by integrating a MoS_2_ layer with TiO_2_ nanowires. The SHG emission from the hybrid material displayed an enhanced signal of more than two orders of magnitude due to the intensified local electric field as shown on Figure 3B. The pattern of SHG varied by the strain field of MoS_2_. The lattice deformation of the MoS_2_ layer induced by the nanowire broke the 3-fold crystal symmetry of MoS_2_ and this deformation led to the anisotropic SHG enhancement. This study illustrates the possibility of tuning SHG intensity and polarization by introducing and adjusting nanowires to the TMDs layer.

Furthermore, Han et al. [62] demonstrated anisotropic enhancement of PL by employing a hybrid system of plasmonic nanowire arrays and WS_2_. WS_2_ flakes were grown through chemical vapor deposition (CVD) and nanowire arrays were fabricated on top using electron-beam lithography and silver metallization. The device was characterized through polarization-resolved PL imaging. The PL images of the TE and TM polarized light showed that the hybrid area of nanowire arrays and bilayer WS_2_ had significant enhancement for both modes at indirect bandgap transition region, when compared to the pristine WS_2_ (Figure 3C).

In addition, Jiang et al. [23] integrated monolayer of WS_2_ flake with plasmonic Au nanorod for PL enhancement. The study designed a heterostructure of a gold nanorod on top of a WS_2_ monolayer. The monolayer was fabricated same as the size of the projected area of the nanorod as can be seen on Figure 3D. The design was to suppress the background PL and to solely monitor the PL of the heterostructure. The study showed PL enhancement compared to that of the pristine monolayer WS_2_. These studies show promising applications on tunable ultrathin photonic devices.

### 4.2 Light guiding

Effective control of radiation properties of light emitters is crucial in developing integrated photonic circuits. Various approaches have been made to make use of these characteristics [63, 64]. For instance, Lin et al. [65] introduced coupled plasmonic waveguide with improved efficiencies that can lighten the trade-off such as confinement loss. Nanowires with 2D materials are also widely used in the field of light guiding.

Zhang et al. [20] theoretically studied and optimized the Kerr nonlinear optical performance of silicon-on-insulator (SOI) nanowire with 2D graphene oxide (GO) film. The study analyzed the influence of the geometry of the waveguide and the thickness of GO film on the propagation loss and nonlinear parameters. The result showed that GO integrated nanowire has the potential of increasing effective nonlinear parameters and enhancing Kerr nonlinearity while minimizing the trade-offs by optimizing the device parameters.

Moreover, Cihan et al. [19] incorporated a silicon nanowire with a MoS_2_ monolayer for the effective control of directionality, polarization state and spectral emission. Conventional Mie theory offers quantifying the scattering contributions from resonances such as electric or magnetic dipoles by Mie coefficient for scattering efficiency. When Kerker conditions are satisfied [66], the directionality is achieved without the need for back reflectors. The study [19] enhanced the directionality by optically coupling a MoS_2_ monolayer with a dielectric antenna. The work grew a MoS_2_ monolayer on a sapphire substrate and drop-casted a tapered (radius 20–40 nm) silicon nanowire on top. Then, the sample is etched using Ar plasma to eliminate any unwanted effect from adjacent bare MoS_2_ regions. The result showed that the top/bottom (T/B) ratio of transverse magnetic polarized emission is more than 20, while that of the bare MoS_2_ is around 0.8. Meanwhile, the T/B ratio image of the hybrid nanowire in transverse electric polarization mode is not clearly distinguishable from its background. This shows that the multipolar resonance in the transverse magnetic polarized mode plays the major role in enhancing the directionality. These studies pave the way of controlling lights on single-photon sources.

## 5 Optoelectronics

Many applications of 1D/2D hybrid materials have been carried out in the field of optoelectronics such as photodetectors, light emitting diodes (LEDs) and phototransistors. Utilization of 1D/2D hybrid materials are one of the attractive viewpoints on these applications. Among these applications, photodetectors have gained a lot of attention in the last few years due to their appealing application in various fields [67], [68], [69]. One of the popular strategies is to implement materials that are functionalized by nanomaterials [70], [71], [72], or to modify their chemical or morphological properties to enhance the output of their applications [73, 74]. Also, using heterojunction of a bulk or nanomaterials is a popular method developed for high-performance photodetectors [75], [76], [77], [78], [79]. These hybrid materials showed noticeable improvement on their responsivity compared to their counterparts. The comparison can be seen on Table 1 [27, 28, 30, 71, 72, 75, 77, 80, 81]. The calculation of responsivity (*R*) is defined by *I*_ph_/(*P* × *A*), where *I*_ph_ is the net photocurrent, *P* represents the incident light intensity, and *A* denotes the effective illuminated area of the detector [27]. The main strategies used on these studies are to enhance the performance of photodetectors by effective charge carrier transfer [30] and light trapping [80].

**Table 1:** Summary of resistivity of nanowire/2D material hybrid materials and their counterparts.

Structure	Bias voltage [V]	Light power density [W/cm^2^]	Light wavelength [nm]	Responsivity [A/W]	Reference
ZnO NWs	1	0.5	405	1.5	[27]
WS_2_ flakes	1	0.5	405	5.03 × 10^−4^	[27]
ZnO/WS_2_ core/shell NWs	1	0.5	405	7.0	[27]
WS_2_ flakes	1	0.5	532	4.84 × 10^−4^	[27]
ZnO/WS_2_ core/shell NWs	1	0.5	532	2.25	[27]
WS_2_ flakes	1	0.5	660	4.58 × 10^−4^	[27]
ZnO/WS_2_ core/shell NWs	1	0.5	660	1.75	[27]
WS_2_ flake	2	–	450	16.7	[30]
WS_2_/CsPbBr_3_ van der Waals plane	2	–	450	57.2	[30]
WS_2_ nanosheet	5	0.01	450	27 × 10^−3^	[28]
CdS NWs/WS_2_ nanosheet	5	0.01	450	3.0	[28]
Cd_3_As_2_ thin film	0.5 × 10^−3^	0.01	650	∼ 6.0 × 10^−3^	[81]
Cd_3_As_2_ thin film/pentacene	0.5 × 10^−3^	0.01	650	36.15 × 10^−3^	[81]
ZnO NR/CuSCN	0	65 × 10^−3^	375	10.9 × 10^−3^	[72]
ZnO NR array/CuSCN/rGO layer	0	65 × 10^−3^	375	18.65 × 10^−3^	[72]
Perovskite layer	5	10^–5^	460	0.008	[80]
Graphene/Au nanostars/perovskites	5	10^–5^	460	5.90 × 10^4^	[80]
Ni/GaN/Ni	10	5 × 10^−6^	365	0.22 × 10^3^	[71]
Ni/Zn-TPPOH/GaN/Zn-TPPOH/Ni	10	5 × 10^−6^	365	4.14 × 10^3^	[71]
TiO_2_ nanotube array	5	2.05 × 10^−3^	350	20.87	[77]
BiOCl nanosheet/TiO_2_ nanotube arrays	5	2.05 × 10^−3^	350	41.94	[77]
Perovskite/graphene	2	0.53 × 10^−6^	660	2.23 × 10^3^	[75]
perovskite/MoS_2_ nanoflakes/rGO	2	0.53 × 10^−6^	660	1.08 × 10^4^	[75]

Nanowires have been widely used on photodetectors for their broad applicability such as employing defects to improve electrical and optical properties [82], [83], [84]. Also, 1D/2D hybrid materials are one of the strategies that are implemented for the optical property enhancement of photodetectors. For example, Wang et al. fabricated [85] GaAs nanowire and GaAs atomic layered sheets as a device material for photodetectors to enhance the responsivity and detectivity. These hybrid materials were able to form a constructive interface state, leading to performance enhancement.

ZnO nanowire has various applications in nanophotonics and optoelectronics, for its structural [86], piezoelectric [87, 88], and optoelectronic properties. However, it has some major issues such as its long response and recovery time. For this reason, Butanovs et al. [27] introduced a ZnO/WS_2_ core–shell hybrid to improve the response time of photodetectors. The study enhanced photodetection by coating the nanowire with a WS_2_ monolayer. WS_2_ monolayers have a distinct property of having a direct bandgap of Δ*E*_dg_ = 1.74–1.79 eV [89, 90] and are strong in optical absorption. In the study [27], the hybrid material was fabricated through deposition of WO_3_ on ZnO nanowires by DC magnetron sputtering of metallic tungsten in the Ar/O_2_ atmosphere and having it annealed at a sulfur atmosphere. The material was later transferred to a gold electrode using a nanomanipulator probe. The response time of the core–shell nanowire at 405 nm was shown to be 55 ms while that of the pristine ZnO nanowire was 5.0 s. ZnO surface is protected by WS_2_ shell from oxygen adsorption and this was shown to influence surface-related photoconducting processes. Density functional theory (DFT) calculations suggested that the WS_2_ shell might have served as a charge carrier channel in the ZnO/WS_2_ heterostructure.

Meanwhile, Gong et al. [28] synthesized CdS nanowires with a WS_2_ nanosheet to enhance the photosensitivity. The hybrid material was synthesized through the CVD method. The responsivity (*R*) was shown to be 3.0 A/W, at a bias voltage of 5 V and incident power of 10 mW/cm^2^, while that of the pristine WS_2_ were 27 mA/W. When the heterojunction is under negative bias voltage, band alignment-induced spontaneous charge carrier transfer results in larger photoresponse properties. Moreover, due to van der Waals epitaxial growth, CdS was easily formed without constraint of WS_2_ lattice. Therefore, by avoiding lattice distortion, photogenerated carriers can transfer efficiently through these interfaces leading to sensitive photodetection.

**Figure 4:**
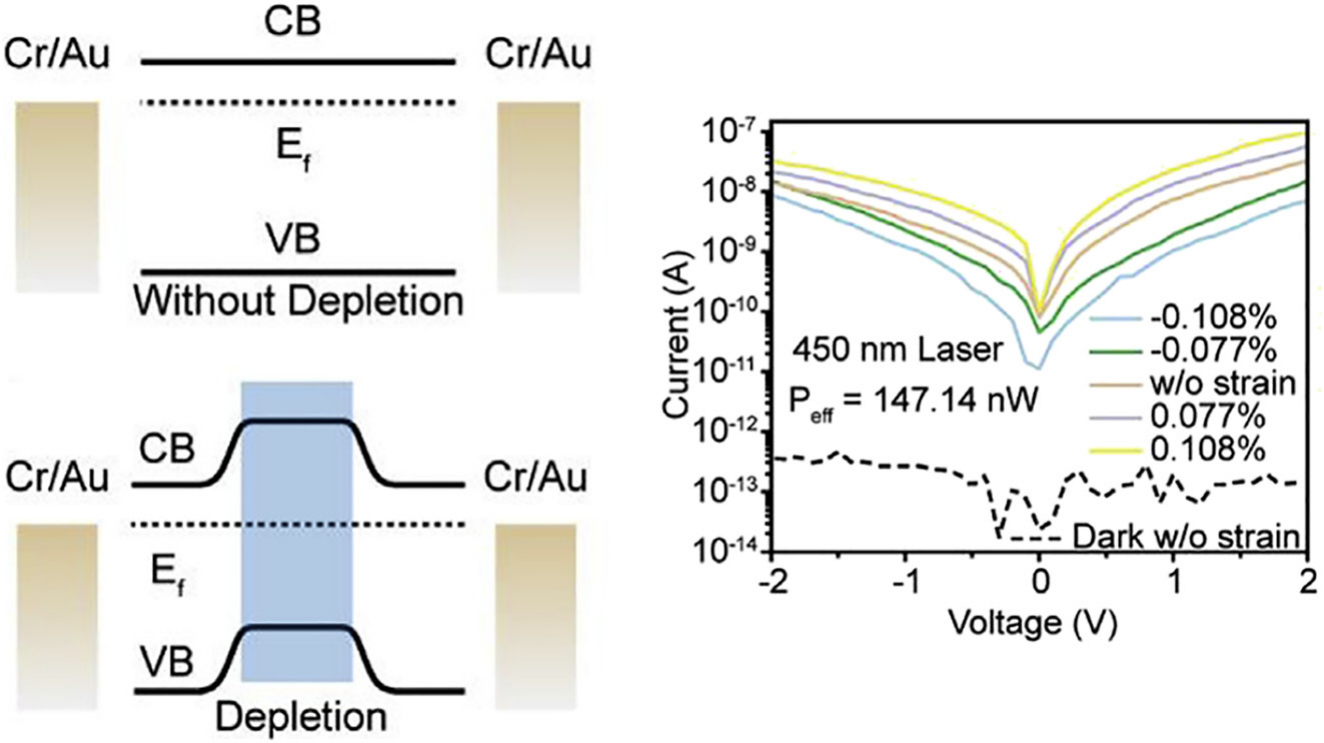
Integration of semiconductor NW and 2D materials for high-performance photodetectors. Left, the band structure of the WS_2_ channel for pristine WS_2_ photodetectors (upper part) and WS_2_/CsPbBr_3_ vdWH photodetectors (lower part), respectively. Right, I–V curves of the WS_2_/CsPbBr_3_ photodetectors under different strains. Reproduced/adapted with permission from Xu et al. [30]. Copyright 2019 Elsevier Ltd.

Furthermore, a high on/off ratio of photodetectors can be achieved by incorporating a nanowire/2D hybrid. Xu et al. [30] used WS_2_ nanoflakes and crystalline CsPbBr_3_ nanowires for developing van der Waals heterostructure (vdWH) photodetectors. The fabricated photodetector exhibited enhanced on/off ratio, responsivity, and detectivity of up to 10^9.83^, 57.2 A/W, and 1.36 × 10^14^ Jones, respectively. When compared with a pure WS_2_ channel, WS_2_/CsPbBr_3_ vdWHs creates a depletion layer as depicted in Figure 4. This leads to suppression of dark current, leading to increase in on/off ratio. In addition, to explore the strain-induced characteristics, the hybrid material was developed on a PEN film. The study showed that the photocurrent increased with tensile strain and decreased with compressive strain (Figure 4) due to the piezo-phototronic effect generated by CsPbBr_3_ nanowires. The high on/off ratio and strain-induced photocurrent open up the possibility of developing high efficiency and multifunctional photodetectors.

Moreover, hybrid materials are often studied on the field of light emitting diode (LEDs). Shen et al. [91] deployed a research on flexible perovskite light emitting diodes (PeLEDs) for enhanced light outcoupling. The research developed a structure of silver nanowire based electrode with crystallized and defect passivated perovskite emitter. The structure led to suppressed nonradiative recombination losses. Combined with outcoupling enhancement by quasi-random nanopatterns of flexible substrate, the study showed external quantum efficiency of 24.5%.

Meanwhile, van der Waals heterostructures of 1D/2D materials have been studied deeply in phototransistors. Jang et al. [92] showed epitaxial structure consisting of AuCN nanowire on a graphene for high responsivity transistor. When photogenerated holes and electrons in AuCN transfer through graphene, extra charges will accumulate in AuCN due to internal potential between AuCN nanowire and graphene interface. This led to tuneable photoresponse based on the applied gate voltage. This facile synthesis method presents a way of optimizing the output of hybrid material by investigating different growth conditions. Furthermore, Qin et al. [93] used dual-channel phototransistor using trigonal selenium nanobelts and ReS_2_ films. The interface between the nanobelts and films improved the separation efficiency of photogenerated electron–hole pairs and this resulted in increased responsivity and detectivity.

## 6 Conclusion and perspectives

Nanowires are shown to be a potential candidate for the application in photonic and optoelectronic devices by incorporating 2D materials. In this review, noble metal nanowires, semiconductor nanowires, and perovskite nanowires are introduced, with their recent application in a conventional application, integrated photonic circuit, light enhancement, path control, and optoelectronics. Moreover, noticeable improvements made by incorporating 2D materials such as TMDs layers, graphene, and graphene oxide are introduced in the review. The studies showed that it is crucial for these 2D materials to be optimized on their structural characteristics such as their sizes, or distance between nanowires. Therefore, in-depth study on optimizing these characteristics is anticipated.

In conclusion, we reviewed nanowires for 2D material-based photonic and optoelectronic devices. Nanowires have potential of usage as a resonator and waveguide in photonic integrated circuits (PICs). Utilizing characteristics of nanowires and using the hybrid of nanowires and 2D materials were introduced. The characteristics and utilizations of different types of nanowires and 2D materials are expected to give fresh perspectives to explore new hybridized materials and ultimately altering the existing devise with enhanced performances.

However, there still are some drawbacks of these hybridizations to overcome. As an example, as they are mixing of nanomaterials, facile synthesis method should be studied. A complicated synthesis method may lead to a low yield rate, be time-consuming, and be relatively high cost. Moreover, their long-term stability still needs to be studied. Severe environments such as high humidity, extremely high or low working temperature could lead to poor performances. Therefore, heightening their repeatability, reproducibility, and experimenting with their performances in a harsh environment is crucial in future developments. Moreover, efforts to enhance the performance of these materials are currently in progress. For example, Yuan et al. [94] developed a fabrication method with increased crystallinity of perovskite nanowires for ultrasensitive photodetectors. Likewise, upcoming applications are anticipated to be on enhancing the outcome by improving the crystallinity of materials and by studying the optimal layout of the devices to realize scalable and integrated systems.
